# A New Treatment for Neuralgia

**Published:** 1885-01

**Authors:** 


### A NEW TREATMENT FOR NEURALGIA.

The latest agent introduced for the relief of neuralgia is a 1 per cent. solution of hyperosmic acid, administered by subcutaneous injection. It has been employed in Billroth's clinic in a few cases. One of the patients had been a martyr to sciatica for years, and had tried innumerable remedies, including the application of electricity no fewer than 200 times, while for a whole year he had adopted vegetarianism. Billroth injected the above remedy between the tuber ischii and trochanter, and within a day or two the pain was greatly relieved, and eventually quite disappeared It would be rash to conclude too much from these results, in the face of the intractability of neuralgiæ to medication, but if it really prove to be as efficacious as considered, hyperosmic acid will be a therapeutic agent of no mean value.

